# TEMPO-Oxidized Cellulose with High Degree of Oxidation

**DOI:** 10.3390/polym9090421

**Published:** 2017-09-06

**Authors:** Zuwu Tang, Wenyan Li, Xinxing Lin, He Xiao, Qingxian Miao, Liulian Huang, Lihui Chen, Hui Wu

**Affiliations:** College of Material Engineering, Fujian Agriculture and Forestry University, Fuzhou 350002, China; 1151045003@fafu.edu.cn (Z.T.); 1161033002@fafu.edu.cn (W.L.); fafuxxl@163.com (X.L.); xiaohe_river@163.com (H.X.); miaoqingxian@163.com (Q.M.); hll65212@163.com (L.H.); lihuichen66@126.com (L.C.)

**Keywords:** cellulose, degree of oxidation, TEMPO

## Abstract

In this paper, water-soluble 2,2,6,6-tetramethylpiperidine-1-oxyl (TEMPO)-oxidized cellulose with a high degree of oxidation was prepared by a two-step process using bamboo dissolving pulp. The first step was to destroy the cellulose crystal I by NaOH/urea solution to obtain cellulose powder with decreased crystallinity. The second step was to oxidize the cellulose powder by TEMPO oxidation. The TEMPO-oxidized cellulose was analyzed by Fourier transform infrared spectroscopy (FTIR), conductimetry, X-ray diffraction (XRD), fiber analyzer, and transmission electron microscopy (TEM). FTIR showed that the hydroxymethyl groups in cellulose chains were converted into carboxyl groups. The degree of oxidation measured by conductimetry titration was as high as 91.0%. The TEMPO-oxidized cellulose was soluble in water for valuable polyelectrolytes and intermediates.

## 1. Introduction

Cellulose is one of the most luxuriant natural macromolecules in the world. As one of the high crystalline structural polysaccharides, cellulose has attracted extensive attention due to its high availability, biodegradability, biocompatibility, and good mechanical properties [1,2]. Cellulose and cellulose derivatives have been widely used in various fields such as paper, textile, coatings, membranes, food additives, sorption agents, flat panel components in liquid crystal displays, and components of medicines [3,4].

Each cellulose is composed of multiple linear (1,4)-linked β-d-glucan chains. However, the hydroxyl groups on one chain form hydrogen bonds with nearby oxygen to construct stable and rigid molecules, making it insoluble in common solvents, which hinder certain applications of cellulose. Various modifications have been developed to improve the solubility and expand the application of cellulose [5,6,7,8,9,10,11,12,13]. For example, through a series of oxidation reaction, aldehyde, carboxyl, and ketone groups can be attached to the cellulose molecular chain, and the modified cellulose exhibits novel functional properties. In particular, 2,2,6,6-tetramethylpiperidine-1-oxyl (TEMPO)-mediated oxidation can produce oxidized cellulose for valuable polyelectrolytes and intermediates, and has attracted a number of investigations [14,15,16,17,18,19,20,21,22,23,24,25,26,27,28]. This technique converts hydroxyl groups at the C6-position of the d-glucose unit into charged carboxyl entities region-selectively. That is, only the hydroxymethyl groups of polysaccharides are oxidized, while the secondary hydroxyls remain unaffected. Individual cellulose nanofibers, 3–4 nm wide and a few microns long, were prepared through a combined TEMPO oxidation and mechanical treatment [15].

The success of using TEMPO/NaBr/NaClO oxidation to produce water-soluble polyglucuronic acid strongly depends on the accessibility and on the crystalline state of the starting material [17,18]. When TEMPO/NaBr/NaClO oxidation is applied to native celluloses with the cellulose I crystal structure, the original fibrous morphologies are unchanged, even after oxidation with sufficient amounts of reagents. Due to the pyknotic inter-molecular and intra-molecular hydrogen bonds, only the surface of the cellulose crystals is selectively oxidized, while the core of the crystals remains intact [14,19]. TEMPO reagents are prevented from entering into the inner cellulose crystals, resulting in a low degree of oxidation of the cellulose.

Bamboo dissolving pulp is a promising alternative non-wood material owing to the high growth rate of bamboo, low resource cost, long or semi-long fibers, and similar α-cellulose content compared to most trees [29]. However, the cellulose I structure in bamboo dissolving pulp makes it unable to dissolve in water and limits its application. Thus, it is desirable to prepare water-soluble TEMPO-oxidized cellulose as an important functional material for a variety of applications.

In this paper, a water-soluble TEMPO-oxidized cellulose with a high degree of oxidation was prepared using bamboo dissolving pulp by a two-step process. During the first step, the cellulose powder with decreased crystallinity was obtained by destroying the cellulose I structure using a NaOH/urea solution. In the second step, cellulose powder was oxidized by TEMPO/NaBr/NaClO to obtain carboxylated cellulose with a high degree of oxidation and high carboxyl content.

## 2. Experimental

### 2.1. Materials

The cellulose was obtained from a bamboo dissolving pulp board, purchased from Sichuan Tianzhu Resources Development Co., Ltd., Sichuan, China. The α-cellulose content of the pulp was 94.5 wt %, the hemicellulose content was 5.2 wt % and the ash content was 0.1 wt %. The weight average molecular weight measured by gel permeation chromatography was 141,000 and the number average molecular weight was 56,000. TEMPO, sodium hypochlorite (NaClO), sodium bromide (NaBr) sodium hydroxide (NaOH), urea, and ethanol were supplied by Sinopharm Group Chemical Reagent Co., Ltd., Tianjin, China. All reagents were analytical grade and used without further purification.

### 2.2. Preparation of TEMPO-Oxidized Cellulose

Ten grams of bamboo dissolving pulp were dissolved into 2 L of NaOH/urea/water solution (7:12:81 by weight) at −12 °C under vigorous stirring [30,31]. After centrifuging at 8000 rpm for 15 min to remove insoluble impurities, the cellulose solution was dropped into 40 L of water. The cellulose suspension was filtered and washed with deionized water repeatedly until neutrality. After cooling in liquid nitrogen for 30 min, the sample was immediately transferred to a freeze-drier and freeze-dried at −50 °C for three days to give NaOH/urea-treated cellulose.

Then, 4.8 g of NaOH/urea-treated cellulose were dispersed in 500 mL of deionized water, and 0.16 g of TEMPO and 12.70 g of NaBr were added. The pH of suspension was controlled at 10 by 0.1 mol/L NaOH solution (monitored with a pH meter). The TEMPO-mediated oxidation of the cellulose powder was initiated by adding 114 mL of 8 wt % NaClO solution dropwise in 10 min and conducted at room temperature under gentle agitation. The solution was reacted for 6 h and the pH was maintained at 10 ± 0.1. The reaction was terminated by adding 5 mL of anhydrous ethanol. The solution was dialyzed in deionized water for two days. The final product of NaOH/urea-treated TEMPO-oxidized cellulose was obtained after freeze-drying.

For comparison, the bamboo dissolving pulp was oxidized directly by TEMPO-mediated oxidization to obtain direct TEMPO-oxidized cellulose [20]. Briefly, 4.8 g of bamboo dissolving pulp was suspended in 500 mL of deionized water containing 0.16 g of TEMPO and 12.70 g of NaBr. The suspension pH was controlled at 10. 114 mL of 8 wt % NaClO solution was dropped into the system in 10 min and reacted for 6 h. After dialyzing for two days, the sample was freeze-dried to obtain direct TEMPO-oxidized cellulose.

### 2.3. Degree of Oxidation (DO)

DO was expressed as the ratio between the amount of oxidized hydroxymethyl groups and total hydroxymethyl groups, which was determined by conductimetric titration. A 50-mg cellulose sample was suspended/dissolved into 15 mL of 0.01 mol/L hydrochloric acid solutions. After 10 min of stirring, the suspension was titrated with 0.005 mol/L NaOH solution under stirring. The conductivity was monitored using a conductivity meter throughout the titration process. The titration was terminated when the pH reached 11. DO was calculated by the following equation [18,19]:

$$\mathrm{DO}=\frac{162\times C\times (V_{2}-V_{1})}{m-36\times C\times (V_{2}-V_{1})}\times 100\%$$

where *C* is the NaOH concentration, *V*_1_ and *V*_2_ are the volume of NaOH, and *m* is the weight of the oven-dried sample.

### 2.4. Fourier Transform Infrared (FTIR) Spectroscopy

Infrared spectra were recorded on an FTIR (BRUKER TENSOR II, Karlsruhe, Germany) spectrometer. Oxidized samples were converted to their acid form in order to displace the carboxyl absorption band toward higher wavelength. The samples were dried in an oven and analyzed as KBr pellets. All spectra were recorded in the wave number ranging from 4000 to 400 cm^−1^ with a resolution of 4 cm^−1^.

### 2.5. Fiber Analyzer

The fiber morphologies of different cellulose samples were investigated by a fiber analyzer (XWY-VIII, Shanghai, China). For this analysis, 0.2 g of the sample was dispersed in 1000 mL ethanol.

### 2.6. Wide Angle X-ray Diffraction (WXRD)

The crystalline structures of different cellulose samples were investigated by a wide angle X-ray diffraction (Shimatzu diffractometer, XRD 6100, Kyushu, Japan) with Cu Kα radiation at the wavelength 1.5405 Å. The continuous scanning angle range used in this study was 5–60°, and the machine was operated at 20 kV and 5 mA. The relative degree of crystallinity was obtained from the crystallinity index (*C*_r_*I*), which could be calculated according to the following equation [32]:

$$C_{r}I=\frac{I_{200}-I_{\mathrm{am}}}{I_{200}}\times 100\%$$

where *I*_200_ is the diffractogram height of (200) peak (2*θ* = 22.6° for cellulose I, 2*θ* = 21.7° for cellulose II), and *I*_am_ is the height of the amorphous background (2*θ* = 19.0° for cellulose I, 2*θ* = 16.0° for cellulose II).

### 2.7. Transmission Electron Microscopy (TEM)

For TEM measurement of NaOH/urea-treated TEMPO-oxidized cellulose, the sample was prepared by depositing 10 μL of 0.025 wt % cellulose solution onto a carbon-film-coated copper grid (500 mesh). The solution was dropped on the grid and dried in air. The TEM measurements were performed using an FEI Tecnai G2 F20 (FEI company, Hillsboro, OR, USA) equipped with a CCD camera (ORIUS SC200, Gatan Inc., Pleasanton, CA, USA) operated at 200 kV.

## 3. Results and Discussion

**Figure 1:**
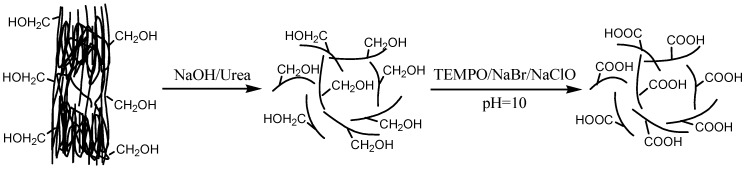
Preparation of NaOH/urea-treated TEMPO-oxidized cellulose using a two-step process.

The NaOH/urea-treated TEMPO-oxidized cellulose with a high degree of oxidation was prepared using a two-step process, as shown in Figure 1. During the first step, the hydrogen bonds in cellulose I are destroyed by NaOH/urea solution, and the individual cellulose chains with decreased crystallinity are obtained. During the second step, highly selective oxidation of the hydroxymethyl groups of individual cellulose to the carboxyl groups was achieved by TEMPO/NaBr/NaClO. NaClO acted as a primary oxidant with catalytic amounts of NaBr and TEMPO. By consuming 2 mol of NaClO, 1 mol of hydroxymethyl group was converted to 1 mol of carboxylate group [14]. In our study, 25.5 mmol of NaClO/g of cellulose was used during TEMPO oxidation, which is much higher than that of the theoretical dosage (12.3 mmol of NaClO/g of cellulose). Therefore, a sufficient amount of NaClO was used for the reaction to obtain TEMPO-oxidized cellulose with a high degree of oxidation.

### 3.1. Infrared Analysis of Oxidized Cellulose

**Figure 2:**
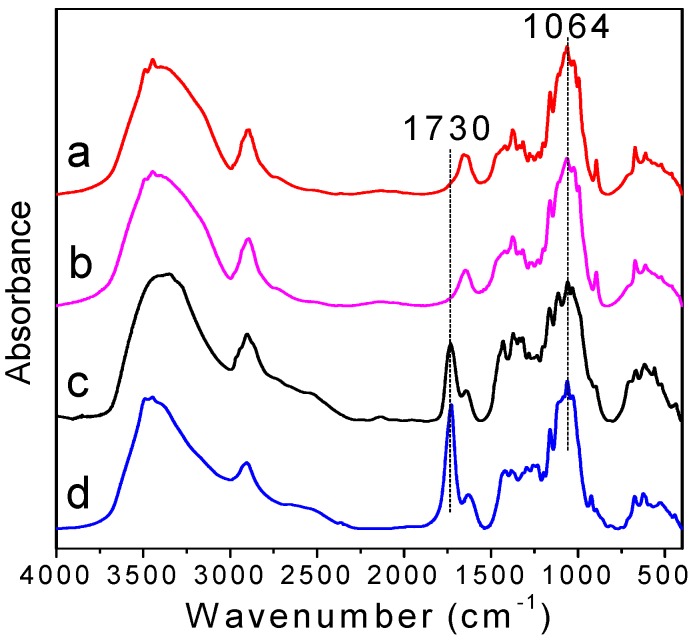
FTIR spectrum of (a) pristine cellulose; (b) NaOH/urea-treated cellulose; (c) direct TEMPO-oxidized cellulose; and (d) NaOH/urea-treated TEMPO-oxidized cellulose.

FTIR spectra of cellulose before and after oxidation were shown in Figure 2. In the spectra of unoxidized cellulose (Figure 2a,b), 1064 cm^−1^ is assigned to C–O stretching of the cellulose backbone and is not influenced by TEMPO-mediated oxidation. This peak can be treated as the internal standard [33,34]. From Figure 2c,d, a new band appears at 1730 cm^−1^, which corresponds to the C=O stretching vibration of carboxyl groups in their acidic form. This suggests that the hydroxymethyl groups of the d-glucose unit were converted into carboxyl groups successfully. The intensity of NaOH/urea-treated TEMPO-oxidized cellulose is stronger than that of direct TEMPO-oxidized cellulose, indicating a higher amount of carboxyl groups and a higher degree of oxidation.

### 3.2. XRD Analysis of Oxidized Cellulose

**Figure 3:**
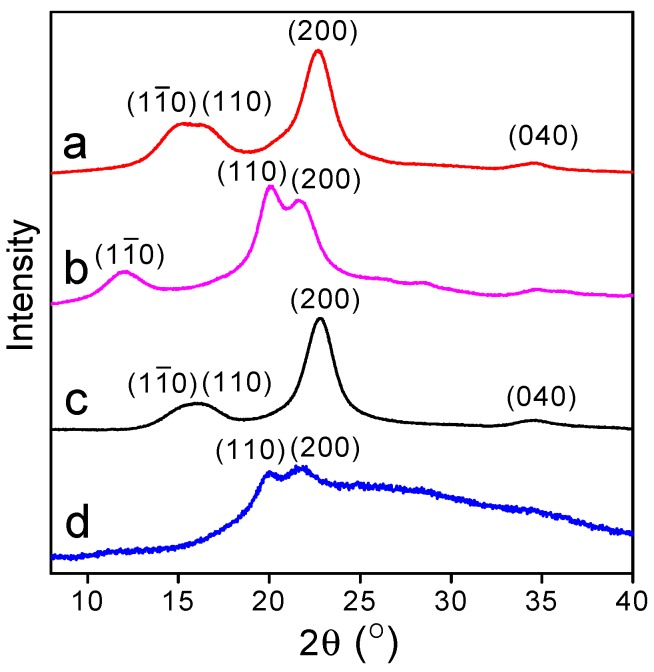
X-ray diffraction curves of (a) pristine cellulose; (b) NaOH/urea-treated cellulose; (c) direct TEMPO-oxidized cellulose; and (d) NaOH/urea-treated TEMPO-oxidized cellulose.

Figure 3a shows the XRD curve of pristine cellulose. The diffraction peaks at 2*θ* = 14.8°, 16.4°, 22.6°, and 34.2° are indexed as ($1\bar{1}0$), (110), (200), and (040) planes [35], respectively, showing the characteristic of cellulose I crystal. After the pristine cellulose was oxidized by TEMPO/NaBr/NaClO (Figure 3b), no significant changes about the peak shape and position were observed, indicating the oxidation resistance of cellulose I crystals. Due to the high crystallinity and low accessibilities to the reagents, the oxidation reaction did not destroy the structure of cellulose I [19]. For the NaOH/urea-treated cellulose (Figure 3c,d), the shape and position of the cellulose diffraction peaks after alkaline treatment changed significantly. The peaks at 2*θ* = 12.3°, 20.2°, and 21.9° are the characteristic of($1\bar{1}0$), (110), and (200) planes of cellulose II crystal [36]. These results indicated that the typical crystalline structure of cellulose I was destroyed and converted into cellulose II during the process of dissolution and regeneration.

The crystallinity of cellulose before and after oxidation was calculated according to the X-ray diffraction pattern, as shown in Table 1. The crystallinity of pristine cellulose is about 71.0%, which is in accordance with the reported results [37,38]. After oxidation, the crystallinity of direct TEMPO-oxidized cellulose becomes 69.5%, which is slightly lower than that of pristine cellulose. The samples in our study were collected by the dialysis process. During the oxidation process, besides the exposed hydroxymethyl groups on the surface of cellulose crystals, some “inner” hydroxymethyl groups partially participated in the oxidation [21]. This may decrease the crystallinity of direct TEMPO-oxidized cellulose. For the pristine cellulose treated with NaOH/urea, the cellulose I crystals were dissolved into NaOH/urea solution and the hydrogen bonds in the cellulose I crystals were destroyed. During the precipitation into water, part of the hydrogen bonds were recovered and cellulose was recrystallized into cellulose II [39]. The crystallinity of NaOH/urea-treated cellulose was reduced to 63.2%. As the NaOH/urea-treated cellulose is further oxidized by the TEMPO/NaBr/NaClO, TEMPO reagents enter and react with the amorphous area and the inner of cellulose II. The resulted crystallinity is only 26.6%.

### 3.3. DO of Oxidized Cellulose

**Figure 4:**
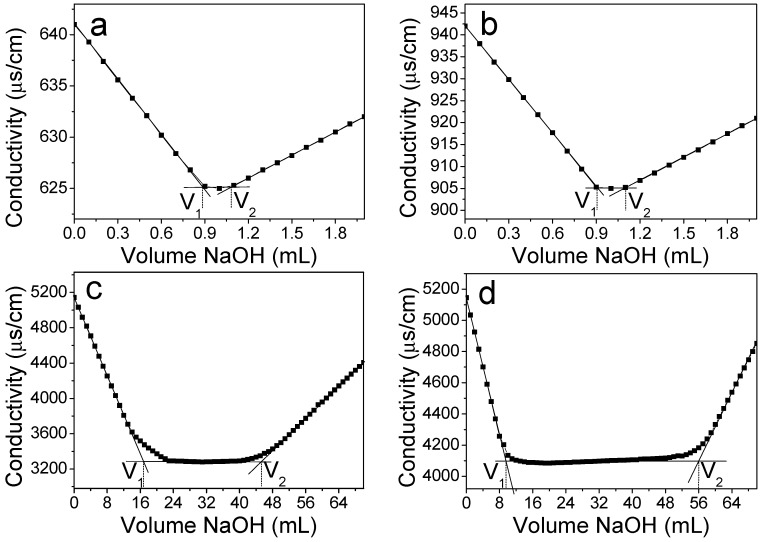
Conductivity titration curve of (**a**) pristine cellulose; (**b**) NaOH/urea-treated cellulose; (**c**) direct TEMPO-oxidized cellulose; and (**d**) NaOH/urea-treated TEMPO-oxidized cellulose.

The DO of cellulose was determined from the conductometric titration. The conductivity versus the volume of NaOH aqueous solution was plotted in Figure 4. The calculated DO of pristine and NaOH/urea-treated cellulose was approximately 0.3%, indicating that trace amounts of carboxyl groups exist in the cellulose. After TEMPO oxidation, the DO of direct TEMPO-oxidized cellulose was 53.0%, showing that about 53.0% of hydroxymethyl groups on pristine cellulose were oxidized into carboxylate groups during TEMPO-mediated oxidation. As expected, the use of sufficient amount of NaClO did not lead to a high DO of direct TEMPO-oxidized cellulose. For the NaOH/urea-treated TEMPO-oxidized cellulose, DO increased significantly to 91.0%. It is reported that the maximum DO of TEMPO-oxidized tunicin whiskers resulting from hydrochloric acid hydrolysis was 10% [19]. The DO of ultrathin cellulose microfibrils extracted from spruce wood powder using combined delignification, TEMPO-catalyzed oxidation, and sonication processes was 50% [21]. In our study, because the NaOH/urea system destroyed the crystal structure of cellulose I, hydroxymethyl groups can be exposed to react with the TEMPO reagent sufficiently. Most of the hydroxymethyl groups were oxidized to carboxyl groups, resulting in a higher DO.

### 3.4. Dispersion States of Oxidized Cellulose

**Figure 5:**
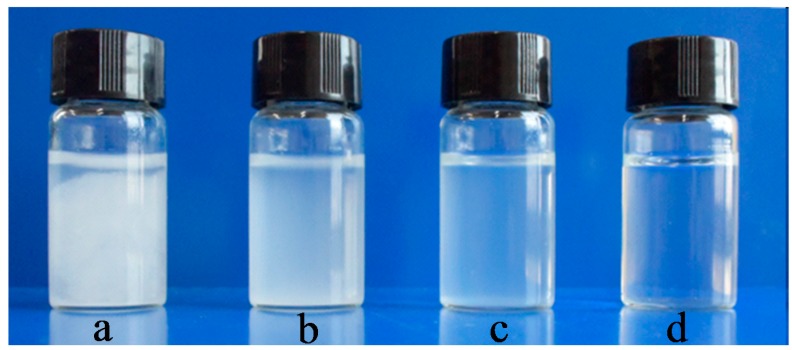
Dispersion states of (**a**) pristine cellulose; (**b**) NaOH/urea-treated cellulose; (**c**) direct TEMPO-oxidized cellulose; and (**d**) NaOH/urea-treated TEMPO-oxidized cellulose.

Figure 5 shows photographs of cellulose before and after oxidation in water. The dispersion states of pristine and NaOH/urea-treated cellulose (Figure 5a,b) are similar. Clear sedimentation was observed at the bottom of the bottle immediately. The direct TEMPO-oxidized cellulose (Figure 5c) could be dispersed in water. After 5 min, sedimentation was also observed. The NaOH/urea-treated TEMPO-oxidized cellulose (Figure 5d) dissolved in water quickly to form transparent solution, showing the good solubility in water. The electrostatic repulsion on the oxidized cellulose facilitates the formation of a uniform aqueous solution.

### 3.5. Morphology

**Figure 6:**
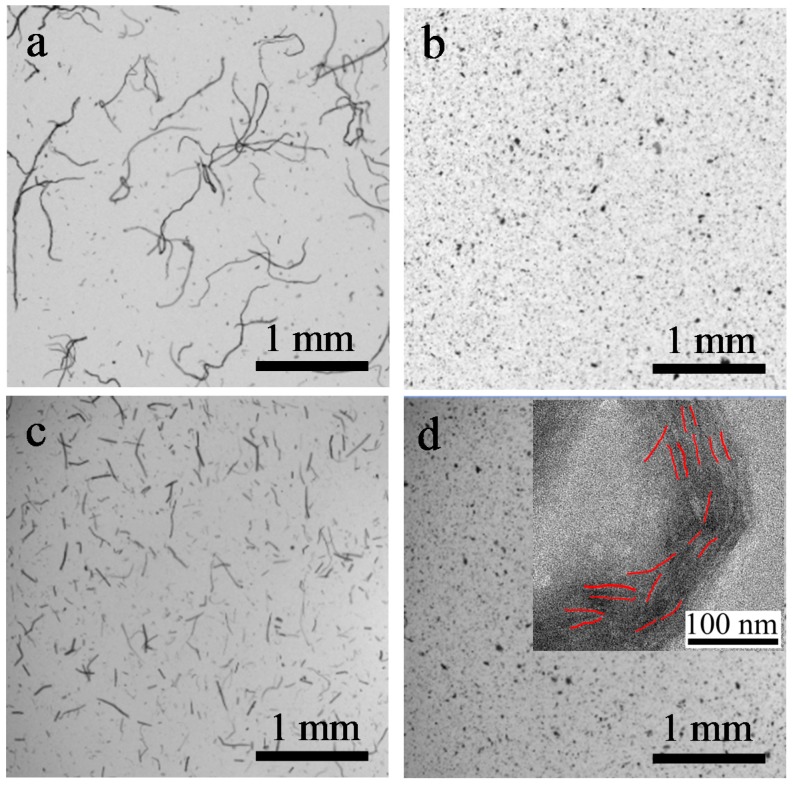
Optical microphotographs of (**a**) pristine cellulose; (**b**) NaOH/urea-treated cellulose; (**c**) direct TEMPO-oxidized cellulose; and (**d**) NaOH/urea-treated TEMPO-oxidized cellulose. Inset in (d) is the TEM image of NaOH/urea-treated TEMPO-oxidized cellulose.

The NaOH/urea-treated TEMPO-oxidized cellulose dissolves in water easily, but not in ethanol. To study the morphology of cellulose before and after oxidation, all the samples were dispersed in ethanol and observed by a fiber analyzer. The pristine cellulose in Figure 6a shows the cellulose bundle morphology. The measured length and width of the cellulose were 1396 ± 5 μm and 16.9 ± 1 μm, respectively. The direct TEMPO-oxidized cellulose in Figure 6c maintained the microfibril shape. Its length was 447 ± 5 μm, which is much shorter than that of pristine cellulose. Because the TEMPO oxidation proceeds from the regions accessible to the reaction with the oxidized TEMPO molecules, the amorphous area was destroyed, and the cellulose bundles were cut into shorter microfibrils. The NaOH/urea-treated cellulose (Figure 6b) and NaOH/urea-treated TEMPO-oxidized cellulose (Figure 6d) presented the morphology of agglomerates. For the NaOH/urea-treated cellulose, when the cellulose was dissolved in the alkaline/urea system and precipitated in water, the dissolved cellulose chains randomly self-assembled and recovered their hydrogen bonded structure to some extent. Thus, the original fiber shape of the pristine cellulose was altered to an irregular powder shape. After oxidation, due to the insolubility in ethanol, NaOH/urea-treated TEMPO-oxidized cellulose also formed agglomerates.

To further determine the dimension of NaOH/urea-treated TEMPO-oxidized cellulose, TEM observation was performed. The inset of Figure 6d shows the TEM image of NaOH/urea-treated TEMPO-oxidized cellulose. We can hardly observe the single TEMPO-oxidized cellulose chains by TEM. However, the visible samples, which are marked in red, exhibited nanostrips with a diameter of 3–5 nm and a length of ca. 37 nm. The length is much shorter and the diameter is comparable with that prepared through a combined TEMPO oxidation and mechanical treatment [15].

## 4. Conclusions

A two-step process was demonstrated to prepare water-soluble TEMPO-oxidized cellulose with a high degree of oxidation using bamboo dissolving pulp as the starting material. After destroying the hydrogen bonds in cellulose I during the first step, the cellulose powder with decreased crystallinity was oxidized by TEMPO/NaBr/NaClO to obtain carboxylated cellulose with a high degree of oxidation during the second step. The TEMPO-oxidized cellulose is soluble in water for valuable polyelectrolytes and intermediates with many possible functionalities.

## Figures and Tables

**Table 1:** Crystallinity index and degree of oxidation of cellulose before and after oxidation.

Samples	Crystallinity Index (%)	Degree of Oxidation (%)
Pristine cellulose	71.0	0.3
Direct TEMPO-oxidized cellulose	69.5	53.0
NaOH/urea-treated cellulose	63.2	0.3
NaOH/urea-treated TEMPO-oxidized cellulose	26.6	91.0
